# For Arthropod Mitochondria, Variety in the Genetic Code Is Standard

**DOI:** 10.1371/journal.pbio.0040175

**Published:** 2006-04-25

**Authors:** Richard Robinson

The protein-making instructions of DNA, and the RNA messages transcribed from them, are spelled out in nucleotides. Proteins, though, are written in amino acids, and one of the seminal discoveries of the early days of molecular biology was the code that relates one to the other. Each of the 20 amino acids is represented by one or more unique RNA triplets, or codons: UAC is decoded as tyrosine, for example, and UGC as cysteine. (U is the RNA nucleotide containing uracil, A is adenine, C is cytosine, and G is guanine.)

For a decade or so after its discovery, the code was believed to be universal, exactly the same in every organism, from bacteria to bonobos. But exceptions—variations in the coding of one or two amino acids—soon turned up, particularly in mitochondria, the subcellular powerhouses in all our cells that have descended from once free-living bacteria. (Mitochondria contain their own DNA and protein-producing machinery, and reproduce independently from the host cell.) Indeed, most of the nonstandard codes discovered to date have been found in the mitochondria of different animal lineages. While there are differences between some animal phyla (chordates, mollusks, and echinoderms, for example), nonstandard mitochondrial codes within an animal phylum have all been considered the same, which has been interpreted to mean that these nonstandard codes arose very early in each lineage and remained unchanged thereafter.

In a new study, Federico Abascal, Rafael Zardoya, and colleagues develop a new analytic technique to show that within one animal phylum—the arthropods—there are two nonstandard codes, and suggest that genetic code changes within a lineage may be more frequent than was earlier believed.

To identify nonstandard mitochondrial genetic codes, the authors compared the mitochondrial coding sequences from 626 different animal species, aligning the sequences to find codons conserved within a gene from one species to the next. They then asked what amino acid any particular codon specified in the protein. The most frequent AA was taken to be the canonical translation of that codon. From there, they could ask whether that same codon is translated as that amino acid in any particular species, in this way identifying potential variant genetic codes. Not every codon position in every gene is conserved between species, of course, and the art of this procedure lies in finding a balance between stringency and tolerance in aligning codons from imperfectly matched sequences. Rigorous exclusion of all misaligned positions produces few but certain data, while a more tolerant approach to mismatches produces more but noisier data. By varying stringency and testing the results against a small set of well-characterized genomes, they arrived at a robust computational approach to analyzing new mitochondrial genomes for nonstandard codons.

They found that while almost every codon translated into the expected amino acid (as deduced from the annotated genetic code) in all species, there was a surprising trend in the arthropods, the largest of all animal phyla, which includes the insects, crustaceans, spiders, and other similar creatures. Among mitochondria from all invertebrates, AGG typically translates as the amino acid serine. Among the 92 mitochondrial genomes from the arthropods, however, AGG coded for serine in 34 species and lysine in 24 other species. Among the rest, the meaning could not be deduced in 18, and 34 species did not use the AGG codon. The authors' analysis of the patterns of change also suggests that the original arthropod mitochondrion used AGG for lysine, not serine.

The sequence of reassignment, disuse, and reversion to the original is difficult to tease out for any lineage within the arthropods, but the variety within the group suggests the code has changed multiple times between the two genetic codes. One explanation for this variety is that pairing of AGG and lysine is disadvantageous for the organism employing it, so that loss or reversion over time would be favored. If true, this explanation suggests there may be multiple other nonstandard codes residing within other lineages that began with a nonstandard and selectively unfavorable coding change. Further application of the authors' analytic method may decode more such surprises in the future.

## 

**Figure:**
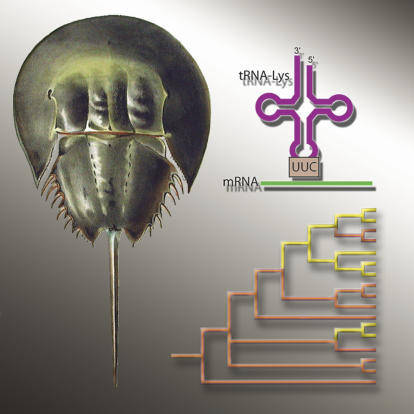
The horseshoe crab uses the newly discovered genetic code (AGG translates into lysine rather than serine). Parallel evolution of this and the typical code of invertebrate mitochondrial genomes (which is correlated with tRNA mutations) occurred repeatedly along the evolutionary history of arthropods. (Figure: horseshoe crab lithograph by George Endicott)

